# Effectiveness of a short video-based educational intervention on factors related to clinical trial participation in adolescents and young adults: a pre-test/post-test design

**DOI:** 10.1186/s13063-018-3097-2

**Published:** 2019-01-03

**Authors:** Joan E. Cowdery, James H. Powell, Yolanda A. Fleming, Devin L. Brown

**Affiliations:** 1 School of Health Promotion and Human Performance, Eastern Michigan University, Ypsilanti, MI 48197 USA; 2 Strategic Medical Associates, Cincinnati, OH 45249 USA; 3 National Medical Association, Project I.M.P.A.C.T, Silver Spring, MD 20910 USA; 4 Stroke Program, University of Michigan, 1500 East Medical Center Drive, Ann Arbor, MI 48109-5855 USA

**Keywords:** adolescent, young adult, video, attitudes, intention, clinical trial participation

## Abstract

**Background:**

Poor clinical trial enrollment continues to be pervasive and is especially problematic among young adults and youth, and among minorities. Efforts to address barriers to enrollment have been predominantly focused on adult diseased populations. Because older adults may already have established attitudes, it is imperative to identify strategies that target adolescents and young adults. The purpose of this study was to test the effectiveness of an educational video on factors related to clinical trial participation among a healthy adolescent and young adult population.

**Methods:**

Participants completed a 49-item pre-test, viewed a 10-min video, and completed a 45-item post-test to assess changes in attitudes, knowledge, self-efficacy, receptivity to, and intention to participate (primary outcome) in clinical trials. Descriptive statistics, paired samples *t*-tests, and Wilcoxon signed-rank tests were conducted.

**Results:**

The final analyses included 935 participants. The mean age was 20.7 years, with almost 70% aged 18 to 20 years. The majority were female (73%), non-Hispanic (92.2%), white (70%), or African American (20%). Participants indicated a higher intention to participate in a clinical trial (*p* < 0.0001) and receptivity to hearing more about a clinical trial (*p* < 0.0001) after seeing the video. Intention to participate (definitely yes and probably yes) increased by an absolute 18% (95% confidence interval 15–22%). There were significant improvements in attitudes, knowledge, and self-efficacy scores for all participants (*p* < 0.0001).

**Conclusions:**

The results of this study showed strong evidence for the effectiveness of a brief intervention on factors related to participation in clinical trials. This supports the use of a brief intervention, in a traditional educational setting, to impact the immediate attitudes, knowledge, self-efficacy, and intention to participate in clinical trial research among diverse, healthy adolescents and young adults.

## Background

Clinical trials are the backbone of medical treatment development and serve as the gold standard by which new treatments are tested. Yet, poor recruitment plagues the majority of trials [1], which slows the pace and increases the cost of medical discoveries. Poor clinical trial enrollment is very common and is a significant challenge across all types of trials and disease areas [1–4]. Adolescents and young adults have the lowest clinical trial participation rates and are least likely to express willingness to consider being in a clinical trial among all age groups [5–7]. Furthermore, minorities of all ages are underrepresented in clinical trials compared with their population representation [8–10].

Because reaching adults with broadly disseminated educational and behavioral interventions about clinical trials has practical limitations, and because some adults may already have established attitudes including longstanding distrust of the medical system [11], it is imperative to identify effective strategies that target adolescents and young adults. High school and college students are on the cusp of adult decision-making age, are easily reachable in an educational setting, and have had fewer opportunities to develop negative attitudes about medical research. Decades of research have established the influence of early attitude formation on attitudes and behaviors throughout the lifespan. Child development and educational psychology research has consistently found that attitudes formed during adolescence persist into adulthood [12–14] and influence subsequent adult behavior, including health behaviors such as diet and exercise [14–16]. Targets to address should include knowledge about clinical trials (the most frequently reported barrier to awareness of clinical trials) and mistrust of research and the medical system (the most frequently reported barrier to acceptance of participation to clinical trials) [13]. Irrespective of level of education, patients offered enrollment into trials struggle with the concept of randomized controlled trials, which suggests that general education of the public about trials is needed [17].

The importance of addressing clinical trial participation among adolescents and young adults is twofold: (1) to prepare them for more immediate clinical trial opportunities that may present, given their already low participation rates and (2) to instill an openness to clinical trial participation that will survive into later adulthood when most trial opportunities are likely to occur. This generates the need to identify predictors of both current and future behavior. The theory of planned Behavior (TPB) posits that behavioral intention is the best predictor of future behavior. The theory aims to predict behavioral intention by identifying an individual’s attitudes, subjective norms, and perceived behavioral control (self-efficacy) [18]. TPB has been widely applied to a multitude of health behaviors in adolescent and young adult populations [19, 20]. Proper application of the model in educational and behavioral interventions requires an understanding of the behavior in the study population [21].

The purpose of this study was, therefore, to test the effectiveness of an existing video regarding clinical trial participation on intention, knowledge, attitudes, self-efficacy, and receptivity to participate in an adolescent and young adult population. If brief, effective strategies were available, widespread educational and behavioral efforts could be undertaken that could ultimately impact future national clinical trial enrollment rates.

## Methods

Undergraduate students at a large public university in southeast Michigan 18 to 40 years of age were offered participation in the study. As a school of opportunity, the 18,000 undergraduate students represent a greater proportion of low-income, part-time, and first-generation undergraduate students than the surrounding institutions. Minorities constitute 30% of the student body while 59% are female. Recruitment was focused on first-year students in introductory general education courses. The effectiveness of the brief intervention was tested with an anonymous pre-test/post-test design. The study was implemented in person during regularly scheduled classes, and electronically through on-line classes or through on-line extra credit opportunities offered within a variety of programs. The study was approved by the University’s Human Subjects Review committee and considered exempt.

### Procedures and data collection

Participants were instructed to complete a 49-item pre-test survey. This consisted of four items to assess participant demographics (age, gender, race, and ethnicity) and a pre-existing set of questions in five domains related to clinical trials: attitudes (20 items), knowledge (13 items), perceived ability/self-efficacy (9 items), receptivity to hear about (1 item), and intention to participate in (1 item) clinical trials [22]. This survey was originally developed to address cancer clinical trials and was previously adapted to include more generic language for presumably healthy individuals [23]. To establish internal consistency of the instrument for a healthy, adolescent and young adult sample, Cronbach’s alpha coefficients were calculated for the 20 attitude and 9 self-efficacy items and were acceptable at 0.84 and 0.91, respectively. One additional question was included to assess willingness to participate in a hypothetical clinical trial (see the Appendix for vignette language).

Upon completion of the pre-test, participants were shown (in person), or directed to (imbedded in the electronic survey), a 10-min video entitled “You’ve Got the Power” created by the National Medical Association as part of Project IMPACT (Increase Minority Participation and Awareness of Clinical Trials) [24]. The goal of the video is to increase awareness, knowledge, and participation of African American adults in biomedical research. Although specifically culturally targeted toward African Americans, the information provided in the video is relevant for all individuals. Immediately after viewing the video, participants completed the 45-item post-test (the same items as the pre-test with the exception of the items on demographics). Each session took approximately 30 min to conduct.

### Outcomes and statistical analysis

The primary outcome of the study was intent to participate in clinical trial research. Secondary outcomes included knowledge, attitudes, self-efficacy, and receptivity regarding clinical trials and clinical trial participation. Participation in a specific hypothetical research trial was investigated as an exploratory outcome.

Descriptive statistics were calculated for the group overall, and by racial and gender subgroups. Subgroup analyses for primary, secondary, and exploratory outcomes were limited to white and African American, female and male, due to the low number of participants in other categories. Intention to participate in and receptivity to hearing more information about clinical trials were each assessed with a single item utilizing a five-point Likert response ranging from “definitely yes” (1) to “definitely no” (5). As these items were measured on an ordinal scale, Wilcoxon signed-rank tests were performed to assess differences in pre- and post-test responses. Responses were also dichotomized into “yes” (“definitely yes” and “probably yes”) and “no” (“unsure,” “probably no,” and “definitely no”), and differences in proportions intent to participate and receptive to hear more about clinical trials between the post-test and pre-test were calculated along with adjusted Wald 95% confidence intervals (CIs).

Knowledge was measured with 13 items with response choices “true,” “false,” and “don’t know.” The knowledge score was calculated as a percentage of correct answers. Attitudes toward clinical trials (positive and negative) were assessed with 20 items, with response choices ranging from “strongly agree” (1) to “strongly disagree” (5). Reversed items were recoded, and a mean scale score was calculated with a range of 1 to 5, with 1 being a more negative attitude about clinical trials. Self-efficacy regarding participants’ perceived ability to make an informed decision about participating in a clinical trial was assessed with 9 items, with response choices ranging from “strongly agree” (1) to “strongly disagree” (5). The self-efficacy mean scale score was calculated with a range of 1 to 5, with 1 indicating higher self-efficacy. Missing values were minimal for the attitude and self-efficacy items and were replaced with the mean value of completed items only if at least 90% of items were completed. Mean differences in scores from pre-test to post-test were calculated for knowledge, attitude, and self-efficacy and compared using paired-samples *t*-tests.

Participation in a hypothetical trial was measured with a single-item Likert response ranging from “very unlikely” (1) to “very likely” (5). Prior to being asked this question, participants were presented with a hypothetical scenario (see the Appendix). Wilcoxon signed-rank tests were performed to assess differences in pre- and post-test responses. Responses were also dichotomized into “yes” (“very likely” and “somewhat likely”) and “no” (“uncertain,” “somewhat unlikely,” and “very unlikely”), and the difference between the proportion likely to enroll at post- and pre-test was calculated along with adjusted Wald 95% CIs. Further analysis was conducted to explore the relationship with post-test responses for participant intention to participate in a clinical trial and actual agreement to participate in the hypothetical trial scenario. The intention item was recoded so that higher responses indicated greater intent to participate. Significance for all analyses was set at 0.05. Analyses were conducted using IBM SPSS 24.0 for Windows, TIBCO Spotfire S+ 8.1 for Windows, and R 3.2.0.

## Results

### Sample and participant characteristics

Of the 1048 participants, 276 submitted print surveys and the remainder were submitted electronically. Of the submitted surveys, 20 participants were eliminated for not being within the 18–40 age range, and 17 for missing the pre-test or the post-test. A question was asked of those who took the test electronically as to whether they watched the entire video. Respondents answering “no” were eliminated (*n* = 86), leaving *n* = 935 available for analysis. The mean age of the sample was 20.7 years (standard deviation 3.69 years), with almost 70% being between 18 and 20 years of age and 96% under the age of 30 (Table 1). The majority of respondents were female (73%) and non-Hispanic (92%). Respondents identifying their race as white comprised 70% of the sample while African Americans accounted for 20%.

**Table 1:** Baseline characteristics of study sample

	Percentage	Number
Age (*n* = 935)		
18–20	67.6	632
21–25	22.9	214
26–29	5.3	50
30–40	4.2	39
Race (*n* = 929)		
White	69.6	647
African American	20.3	189
American Indian/Alaskan Native	0.3	3
Asian	3.1	29
Native Hawaiian/Other Pacific Islander	0.2	2
More than one race	6.4	59
Ethnicity (*n* = 915)		
Of Hispanic origin	7.8	71
Not of Hispanic origin	92.2	844
Gender (*n* = 934)		
Female	73.1	683
Male	26.2	245
Transgender	0.6	6

### Primary outcome

Participants indicated a higher level of intention to participate (49.0% to 67.9%) in a clinical trial after seeing the video (*Z* = −8.70, *p* < 0.0001). This result was consistent both among men and women and among African Americans and whites (*p* < 0.0001; Table 2). Similarly, the proportion with intent to participate was higher in the post-test (49% vs 68%, difference = 18%; 95% CI 15–22%).

**Table 2:** Pre- and post-test receptivity, intention, and likelihood of participating in clinical trials

	Pre-test			Post-test			Difference in proportions** (95% CI)	
	*n*	Median	IQR	*n*	Median	IQR		p***
Receptivity*								
Total sample	931	2	1.00, 2.00	933	1	1.00, 2.00	0.06 (0.03, 0.08)	< 0.0001
White	646	2	1.00, 2.00	646	1	1.00, 2.00	0.05 (0.02, 0.07)	< 0.0001
African American	187	2	1.00, 3.00	189	2	1.00, 2.00	0.11 (0.04, 0.18)	0.02
Intention*								
Total sample	930	3	2.00, 3.00	932	2	2.00, 3.00	0.18 (0.15, 0.22)	< 0.0001
White	645	3	2.00, 3.00	646	2	2.00, 3.00	0.20 (0.16, 0.24)	< 0.0001
African American	187	2	2.00, 3.00	188	2	2.00, 3.00	0.13 (0.05, 0.22)	0.03
Participate in hypothetical trial*								
Total sample	930	3	2.00, 4.00	929	4	2.00, 4.00	0.15 (0.12, 0.18)	< 0.0001
White	645	3	2.00, 4.00	644	4	3.00, 4.00	0.14 (0.10, 0.17)	< 0.0001
African American	187	3	2.00, 4.00	187	3	2.00, 4.00	0.16 (0.08, 0.23)	< 0.0001

*CI* confidence interval, *IQR* interquartile range

*Lower scores indicate greater receptivity to hearing more about a relevant clinical trial, greater intention to participate, and lower likelihood of participation in a hypothetical clinical trial

**Proportion receptive to hearing more about clinical trials, intent to participate, and likely to enroll in a hypothetical clinical trial: difference between dichotomous post- and pre-test values along with adjusted Wald 95% confidence intervals

***From Wilcoxon signed-rank test applied to pre-test and post-test comparison

### Secondary outcomes

Participants indicated significantly higher receptivity to hearing more about a clinical trial after watching the video (*Z* = −8.415, *p* < 0.0001). Significant increases in receptivity were found both among men and women and among African Americans and whites (*p* < 0.0001; Table 2). Similarly, the proportion receptive to hearing more about clinical trials was higher in the post-test (82% vs 89%, difference = 6%; 95% CI 3–8%).

Comparison of mean pre- and post-test scores for knowledge, attitude, and self-efficacy are presented in Table 3. For all participants, there were significant improvements in mean pre- and post-test scores for knowledge (*t*(933) = 16.27, *p* < 0.0001), attitude (*t*(931) = 16.51, *p* < 0.0001), and self-efficacy (*t*(930) = 16.17, *p* < 0.0001). Significant increases in knowledge (*t*(681) = 13.88 and *t*(244) = 8.24), positive attitudes (*t*(679) = 13.93 and *t*(244) = 8.82), and self-efficacy (*t*(679) = 15.07 and (*t*(243) = 6.25) were identified for female and male participants, respectively (all *p* < 0.0001). Significant positive differences in knowledge, attitude, and self-efficacy between pre- and post-test scores were identified for both white and African American participants as well (all *p* < 0.0001; Table 3).

**Table 3:** Pre- and post-test scale scores and their comparisons, for knowledge, attitude, and self-efficacy: all participants and among racial subgroups

	*n*	Pre-test	Post-test	Mean difference	p**
		mean (SD)	mean (SD)	(95% CI)	
All participants					
Knowledge*	934	58.03 (20.47)	68.47 (19.16)	10.44 (9.18, 11.70)	< 0.0001
Attitude*	932	3.16 (0.42)	3.41 (0.54)	0.25 (0.23, 0.28)	< 0.0001
Self-Efficacy*	931	2.05 (0.62)	1.77 (0.69)	−0.28 (− 0.25, − 0.31)	< 0.0001
Whites					
Knowledge*	647	61.12 (18.58)	71.21 (17.46)	10.09 (8.69, 11.50)	< 0.0001
Attitude*	647	3.20 (0.42)	3.46 (0.52)	0.26 (0.23, 0.30)	< 0.0001
Self-Efficacy*	647	2.02 (0.58)	1.72 (0.67)	−0.30 (− 0.26, − 0.34)	< 0.0001
African Americans					
Knowledge*	189	49.65 (23.21)	62.07 (20.52)	12.41 (9.08, 15.75)	< 0.0001
Attitude*	187	3.06 (0.37)	3.23 (0.57)	0.16 (0.09, 0.24)	< 0.0001
Self-Efficacy*	186	2.11 (0.67)	1.92 (0.70)	−0.20 (− 0.12, − 0.28)	< 0.0001

*CI* confidence interval, *SD* standard deviation

*Higher scores indicate greater knowledge, more favorable attitude, and lower self-efficacy

**From Wilcoxon signed-rank test applied to pre-test and post-test comparison

### Exploratory outcome

When presented with a hypothetical scenario, participants were more likely to agree to be in the trial after viewing the video, as a whole, both among men and women and among African Americans and whites (*p* < 0.0001; Table 2). The proportion likely to enroll in the hypothetical trial was higher in the post-test (40% vs 56%, difference = 15%; 95% CI: 12–18%). Responses to the intention to participate question (primary outcome) and the response to the hypothetical trial scenario (both post-test) were associated (*Z* = − 15.79, *p* < 0.001; Table 4).

**Table 4:** Intention to participate by hypothetical trial participation cross-tabulation

		Participate in hypothetical trial				
		Very unlikely	Somewhat unlikely	Uncertain	Somewhat likely	Very likely
Intention to participate	Definitely no	6	0	5	0	2
	Probably no	12	19	7	5	0
	Unsure	25	36	94	75	12
	Probably yes	32	76	62	243	65
	Definitely yes	13	15	8	55	61

Wilcoxon signed-rank test (*Z* = −15.79, *p* < 0.0001)

## Discussion

This single-group pre-test/post-test intervention shows strong evidence for the effectiveness of a brief video on factors related to participation in clinical trial research in an adolescent and young adult population. The impact of the intervention on the primary outcome was evidenced by significant increases in intention to participate in clinical trial research in general, and indirectly supported by the significant increase in intent to participate in the specific trial described in the hypothetical scenario. Secondary outcome measures of knowledge, attitude, self-efficacy regarding clinical trials, and receptivity to hearing more about clinical trials also showed significant increases from pre-test to post-test scores.

Previous studies have shown that brief educational interventions can be effective in increasing knowledge of clinical trials [25, 26]. Research has also demonstrated that brief educational efforts can impact attitudes and willingness to participate in clinical trial research [22, 27]. However, previous studies have focused almost exclusively on adult cancer patient populations. To our knowledge, this is the first study to explore the effectiveness of a brief video intervention on factors related to hypothetical clinical trial participation in a healthy adolescent and young adult population in a traditional educational setting. Furthermore, the results of this study show the effectiveness of a culturally targeted intervention among young African Americans. This is particularly relevant given the documented differences in attitudes and participation rates in clinical trials between African American and non-Hispanic white patient populations [28]. The study also supports the effectiveness of this intervention in the majority population, since there was not a large nominal difference between groups, despite the racial targeting of the video. This supports a cost-saving approach of developing a single intervention targeted towards a higher risk minority population that will also be effective in the majority population.

Although we were unable to validate behavioral intent against actual enrollment, we did assess the association between behavioral intent to enroll in a trial and response to enrollment in a hypothetical trial. These two were highly associated. The benefits of addressing the topic of clinical trials in a presumably healthy population are vast, but application to this population does preclude testing the effects of the intervention on enrollment into an actual clinical trial for a disease state relevant to the individual participants.

Limitations of this study include the use of a video designed for an African American adult population. Although those who participated electronically were asked a question to determine whether they watched the entire video, we were not able to determine the level of engagement with the video for electronic or face-to-face participants. However, the increase in post-test knowledge helps validate participant engagement. Furthermore, although small in number, those who participated electronically and indicated they did not watch the video were excluded from the analysis. This may have contributed to a slight over-estimate of the effectiveness of the intervention. It is unclear from this brief study as to the durability of the results and how well intention to participate in clinical trial research will translate to future real-world scenarios if and when the individual were to become ill and were offered enrollment in a clinical trial. Generalizability is limited due to the use of a single university with a mostly regional population as the sample. Finally, this single-group pre-test/post-test design lacked a control group and thus, has limited external validity. The immediacy of the post-test should mitigate this somewhat, however.

## Conclusions

The results of this study support the use of a brief intervention, designed for a general African American adult population, in a traditional educational setting to impact the immediate attitudes, knowledge, self-efficacy, and intention to participate in clinical trial research among diverse, healthy late adolescents and young adults. The next steps should include the development and longitudinal testing of an age-appropriate, culturally relevant intervention in a younger adolescent population in an educational setting. If such an approach were deemed effective, widescale dissemination through a program embedded in the high school curriculum could influence schoolchildren nationwide and ultimately significantly impact clinical trial participation for years to come.

## Appendix

Pretend you have a condition called asthma (“az-ma”). This causes problems with breathing. You miss school, sports, and social things about 5 days a month because of your breathing. You already use inhalers and are followed by a doctor. Because your asthma is still a problem for you, your doctor asks if you want to be in a clinical trial. Here’s more information: The trial compares a new pill to a “dummy pill” called a “placebo” (pill that has no action). The study will test whether the new pill helps teens with asthma. Small studies have suggested that the new pill may be helpful. But, there may be side effects. Few people who take the new pill get some type of infection. This infection may require medicine to treat it. Sometimes the infection can be serious. A computer will assign you to get either the new pill or the dummy pill. You have equal chances of getting the new pill and the dummy pill – like flipping a coin. You and your doctor won’t know which pill you are using. They both look the same. You will take the pill once a day for 6 months. You will keep track of the days you miss activities because of your breathing. You will turn this information in at your doctor visits.

How likely would you be to be in this trial? Very unlikely, Somewhat unlikely, Uncertain, Somewhat likely, Very likely.

## Abbreviations

CI: Confidence interval
IMPACT: Increase Minority Participation and awareness of Clinical Trials
IQR: Interquartile range
TPB: Theory of planned behavior
